# Supplementary material for: The Evolution and Characterization of the RNA Interference Pathways in Lophotrochozoa
Source: Genome Biol Evol. 2024 May 7;16(5):evae098. doi: 10.1093/gbe/evae098 (PMC11114477; doi:10.1093/gbe/evae098)
Supplement: evae098_Supplementary_Data [file evae098_supplementary_data.zip › Supplementary_materials.zip!/selected_miRNAs/NoGe_selected_miRNAs/mirdeep_pdf_files/NMRB01000004.1_Notospermus_geniculatus_strain_Ushimado_scaffold4,_whole_genome_shotgun_sequence_157.pdf]

Provisional ID	:	NMRB01000004.1_Notospermus_geniculatus_strain_Ushimado_scaffold4,_whole_genome_shotgun_sequence_157
Score total	:	769.4
Score for star read(s)	:	3.9
Score for read counts	:	764.9
Score for mfe	:	1.3
Score for randfold	:	0
Score for cons. seed	:	-0.6
Total read count	:	1512
Mature read count	:	1506
Loop read count	:	0
Star read count	:	6

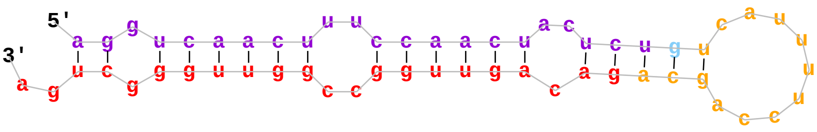

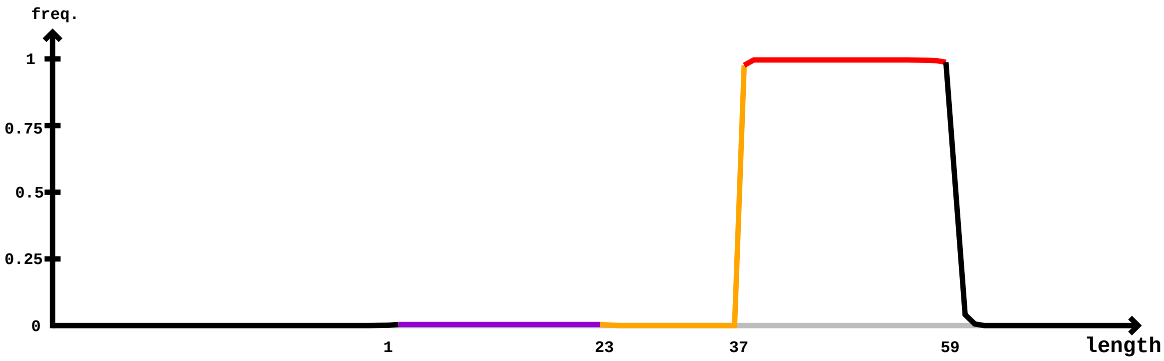


```
                                  Star                                 Mature
5'- guugugagggucaaagugaaugguuugugacauaaggucaacuuuccaacuacucugucauuuuccagcagacaguuggccgguugggcugauguuuacagaucagugcau   -3'    obs
    guugugagggucaaagugaaugguuugugacauaaggucaacuuuccaacuacucugucauuuuccagcagacaguuggccgguugggcugauguuuacagaucagugcau          exp
    ...............(((..(((((((((((((.((.((((((..((((((..(((((.........))))).))))))..)))))).)).))).)))))))))).)))..    reads     mm     sample
    ......................................................................gacaguuggccgguugggcu.....................    2         0      Fm3
    ......................................................................gacaguuggccgguuggAcug....................    1         1      Fm3
    ......................................................................Uacaguuggccgguugggcuga...................    1         1      Fm3
    ......................................................................gCcaguuggccgguugggcuga...................    1         1      Fm3
    ......................................................................gacaguuggccgguuggAcuga...................    3         1      Fm3
    ......................................................................gacaguuggccgguuUggcuga...................    2         1      Fm3
    ......................................................................gacaguuggccgguugCgcuga...................    3         1      Fm3
    ......................................................................Nacaguuggccgguugggcuga...................    1         1      Fm3
    ......................................................................gacaguuggccgguugggAuga...................    1         1      Fm3
    ......................................................................gacaguuggccggAugggcuga...................    1         1      Fm3
    ......................................................................gacaguuggccgguugggcugU...................    1         1      Fm3
    ......................................................................gacaguuggccgguugAgcuga...................    4         1      Fm3
    ......................................................................gacaguuggccgguuggUcuga...................    1         1      Fm3
    ......................................................................gacaguuggGcgguugggcuga...................    1         1      Fm3
    ......................................................................gacagAuggccgguugggcuga...................    1         1      Fm3
    ......................................................................gacaguuggcAgguugggcuga...................    1         1      Fm3
    ......................................................................gacaguuggccgguuggCcuga...................    3         1      Fm3
    ......................................................................gacaguuggcGgguugggcuga...................    2         1      Fm3
    ......................................................................gacaguuggccgguugggcuga...................    524       0      Fm3
    ......................................................................gacaguuggccgguugggcugau..................    5         0      Fm3
    ......................................................................gacaguuggccgguugggcugaA..................    16        1      Fm3
    ......................................................................gacaguuggccgguugggcugUu..................    1         1      Fm3
    ......................................................................gacaguuggccgguugggcugauA.................    4         1      Fm3
    .......................................................................acaguuggccgguugggGuga...................    1         1      Fm3
    .......................................................................acaguuggccgguugggcuga...................    10        0      Fm3
    .......................................................................acaguuggccgguugggcugau..................    1         0      Fm3

    ......................................................................gaAaguuggccgguugggcuga...................    1         1      Fm1
    ......................................................................gacaguuggccgguugggcuga...................    203       0      Fm1
    ......................................................................gacaguuggUcgguugggcuga...................    1         1      Fm1
    ......................................................................gacaguuggccgguugggcuAa...................    1         1      Fm1
    ......................................................................gacaguuggccgguugCgcuga...................    1         1      Fm1
    ......................................................................gacaguuggccgguugggcugU...................    2         1      Fm1
```

```
                                   Star                        Mature
guugugagggucaaagugaauggguuugugacauaaggucaacuuuccaacuacucugucauuuuccagcagacaguuggccgguugggcugauguuuacagaucagugcau
.......................................................................gacGguuggccgguugggcuga...................   1    1    Fm1
.......................................................................gacaguuggccgguugggcugG...................   4    1    Fm1
.......................................................................gacaguuggccgguuggCcuga...................   1    1    Fm1
.......................................................................gacaAuuggccgguugggcuga...................   1    1    Fm1
.......................................................................gacaguuggccgguuggAcuga...................   3    1    Fm1
.......................................................................gacaguuggccgguugggcugau..................   23   0    Fm1
.......................................................................gacaguuggccgguugggcugaA..................   5    1    Fm1
.......................................................................gacaguuggccgguugggcugauU.................   8    1    Fm1
.......................................................................gacaguuggccgguugggcugauA.................   4    1    Fm1
........................................................................acaguuggccgguugggcuga...................   2    0    Fm1
........................................................................acaguuggccgguugggcugaA..................   3    1    Fm1

.......................................................................Uacaguuggccgguugggcuga...................   1    1    Fm2
.......................................................................gacaguuggccgguugAgcuga...................   1    1    Fm2
.......................................................................gacaguuggccgguuggAcuga...................   1    1    Fm2
.......................................................................gacaguuggccgguugggcuga...................   242  0    Fm2
.......................................................................gacaguuggcGgguugggcuga...................   1    1    Fm2
.......................................................................gacaguuggccgguugggcugG...................   10   1    Fm2
.......................................................................gacaguuggccgguuggUcuga...................   2    1    Fm2
.......................................................................gacaguuggccgguugggcugaA..................   25   1    Fm2
.......................................................................gacaguuggccgguugggcugau..................   11   0    Fm2
.......................................................................gacaguuggccgguugggcugauU.................   15   1    Fm2
.......................................................................gacaguuggccgguugggcugauA.................   6    1    Fm2
........................................................................acaguuggccgguugggcuga...................   5    0    Fm2
........................................................................acaguuggccgguugggcugau..................   3    0    Fm2

.......................................................................gacaguuggccgguugggcugU...................   3    1    Mm3
.......................................................................gacaguuggccgguugggcugG...................   1    1    Mm3
.......................................................................gacaguuggccgguugggcuga...................   34   0    Mm3
.......................................................................gacaguuggccgguugggcugaA..................   2    1    Mm3
.......................................................................gacaguuggccgguugggcugau..................   6    0    Mm3
.......................................................................gacaguuggccgguugggcugauU.................   1    1    Mm3

.................................uaaggucaacuuuccaacuacucu.......................................................   1    0    Mm2
..................................aaggucaacuuuccaacuacucu.......................................................   1    0    Mm2
...................................aggucaacuuuccaacuacucu.......................................................   4    0    Mm2
.......................................................................gacaguuggccgguuggg.......................   1    0    Mm2
.......................................................................gacaguuggccgguugggc......................   1    0    Mm2
.......................................................................gacaguuggccgguugggcug....................   2    0    Mm2
.......................................................................gacaguuggccgguugggcugU...................   2    1    Mm2
.......................................................................gacaguuggcAgguugggcuga...................   1    1    Mm2
.......................................................................gacaguuggccgguugggcuga...................   107  0    Mm2
.......................................................................gacaguuggccgguugggcugG...................   4    1    Mm2
.......................................................................gacaguuggccgguugggcugUu..................   1    1    Mm2
.......................................................................gacaguuggccgguugggcugau..................   6    0    Mm2
.......................................................................gacaguuggccgguugggcugaA..................   10   1    Mm2
.......................................................................gacaguuggccgguugggcugaG..................   1    1    Mm2
.......................................................................gacaguuggccgguugggcugauA.................   4    1    Mm2
.......................................................................gacaguuggccgguugggcugauU.................   5    1    Mm2
........................................................................acaguuggccgguugggcuga...................   4    0    Mm2
........................................................................acaguuggccgguugggcugau..................   1    0    Mm2

.......................................................................gacaguuggccgguugggGug....................   1    1    Mm1
.......................................................................gacaguuggccgguugggcug....................   5    0    Mm1
.......................................................................gacaguuggccgguugCgcuga...................   1    1    Mm1
.......................................................................gacaguuggccgguugggcuga...................   85   0    Mm1
.......................................................................gacaguuggccgguugggcugU...................   2    1    Mm1
.......................................................................gacaguuggccgguuggUcuga...................   1    1    Mm1
.......................................................................gacaguuggccgguugUgcuga...................   1    1    Mm1
.......................................................................gacaguuggccgguuggAcuga...................   1    1    Mm1
.......................................................................gacaguuggccgguugggcugaA..................   14   1    Mm1
.......................................................................gacaguuggccgguugggcugau..................   2    0    Mm1
.......................................................................gacaguuggccgguugggcugaG..................   2    1    Mm1
.......................................................................gacaguuggccgguugggcugauU.................   6    1    Mm1
.......................................................................gacaguuggccgguugggcugaAgu................   9    1    Mm1
```
